# Daratumumab induced lichenoid drug eruption

**DOI:** 10.1016/j.jdcr.2026.02.004

**Published:** 2026-02-10

**Authors:** Curtis Perz, Thomas Wissman, Aubrey Montebello

**Affiliations:** a Dermatology Residency Program, San Antonio Uniformed Services Health Education Consortium, San Antonio, Texas; b Philadelphia College of Osteopathic Medicine, Philadelphia, Pennsylvania

**Keywords:** Amyloidosis, Daratumumab, Lichenoid drug eruption, Multiple myeloma, Triamcinolone acetonide acetate

## Introduction

Currently, there are no reports of daratumumab induced lichenoid drug eruption in the literature. Reports of adverse cutaneous reactions secondary to daratumumab in clinical trials and real-world pharmacovigilance studies have been limited to local injection-site reactions and other non-specific descriptions such as rash, urticaria, and pruritus.^1^, ^2^, ^3^, ^4^, ^5^ This includes the ANDROMEDA trial, a phase III study which helped establish daratumumab-based regimens as a first-line treatment for newly diagnosed light-chain amyloidosis, as in our patient. This trial reported local injection-site reaction as the main adverse cutaneous reaction, occurring in 10.9% of patients with grade 1 or 2 severity, with no significant adverse cutaneous reactions identified.^5^ Additionally, there are a few reports that highlight non-specific rash in association with systemic symptoms following daratumumab infusion.^1^^,^^6^

Lichenoid drug eruption is an uncommon type of cutaneous adverse drug reaction that can clinically manifest weeks to years after initiation of a culprit medication.^7^^,^^8^ The typical presentation consists of erythematous-to-violaceous scaly papules and plaques present symmetrically on the trunk and extremities with pruritus. Involvement of the oral mucosa is not common, but can occur in isolation. Those affected are typically middle-aged or older and there is no sex predilection. Known culprit medications include thiazide diuretics, beta-blockers, immune checkpoint inhibitors, and anti-tumor necrosis factor-α monoclonal antibodies, among others. Lichenoid drug eruption clinically and histologically mimics idiopathic lichen planus. Clinical features that favor lichenoid drug eruption include a widespread or photodistributed pattern, extensor surface involvement, pronounced scaling, lack of wickham striae, and sparing of the genitals or oral mucosa.

## Case report

We present the case of a 48-year-old Fitzpatrick IV male with a history of multiple myeloma status post autologous stem cell transplant and AL amyloidosis who presented with a waxing and waning rash over the course of 15 months. The rash started 6 months after he completed a total of 6 cycles of daratumumab for the treatment of AL amyloidosis and multiple myeloma. He initially presented to dermatology with an abrupt onset, erythematous rash on the chest and back that was notably asymptomatic otherwise. A punch biopsy at that time exhibited lichenoid and perivascular dermatitis with erythrocyte extravasation. Though a small, indeterminate amyloid-like focus was observed, it was deemed likely incidental and insufficient for subtyping. Treatment with topical steroids provided minimal relief.

**Fig 1:**
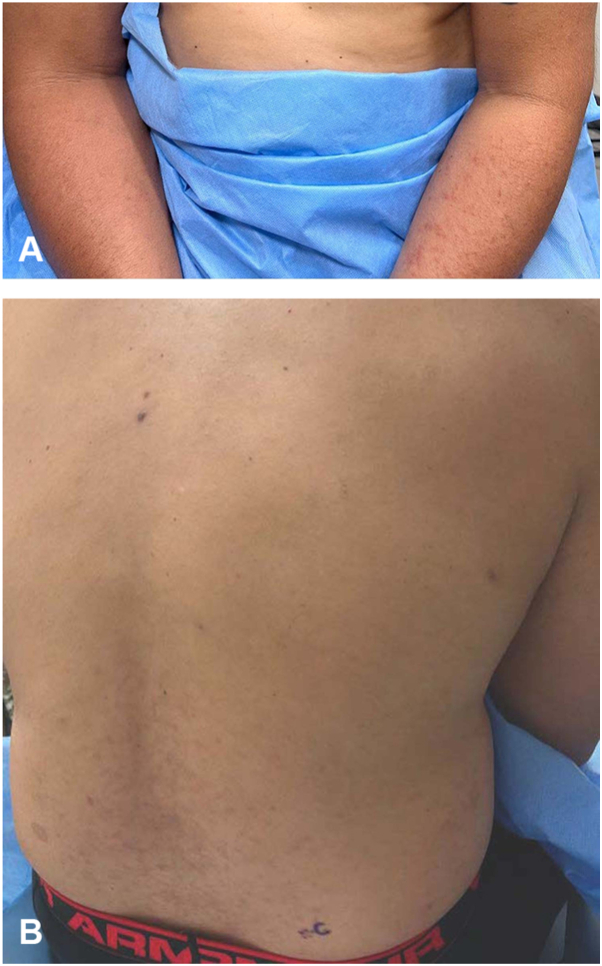
Clinical findings: **A,** Diffuse, *skin colored* to violaceous papules coalescing into plaques on the dorsal and ventral forearms. **B,** Violaceous papules and plaques on the lower back extending to the flanks.

Just over a year later, he presented with widespread progression of the rash involving the trunk and extremities. At this point, he had completed 20 cycles of daratumumab. Unlike his previous rash, this time he endorsed severe pruritus. He otherwise denied systemic symptoms or new medications. Physical exam revealed numerous 4 to 6 mm red-violaceous papules coalescing into thin plaques on the abdomen, back and dorsal forearms (Fig 1, *A* and *B*). There was no oral involvement.

**Fig 2:**
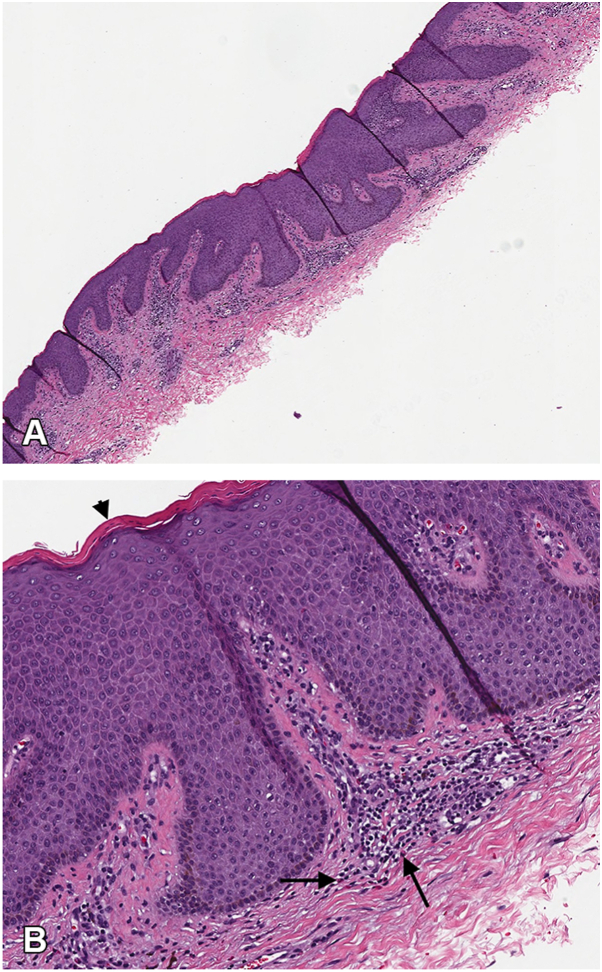
Pathology findings: **A,** (H&E, 10×), Parakeratosis, acanthosis and lichenoid infiltrate appreciable at lower power. **B,** (H&E, 40×), The *arrowhead* exhibits focal parakeratosis. The *larger arrows* point out eosinophils in the lichenoid infiltrate, which can be seen tracking upwards into dermal papillae. *H&E*, Hematoxylin & Eosin.

Shave biopsy of the lower back revealed lichenoid interface dermatitis and scattered eosinophils (Fig 2, *A* and *B*), likely suggestive of a dermal hypersensitivity reaction. There was no evidence of fungal organisms, vasculitis, granulomatous inflammation, or malignancy. Given the intensity of pruritus and rash burden, progression of lichenoid drug eruption secondary to daratumumab was favored. We treated him in clinic with 1 mL of intramuscular (IM) triamcinolone acetonide acetate 40 mg/mL to alleviate pruritus, then prescribed topical triamcinolone for rash treatment. His pruritus significantly improved in a few days, and the rash had completely resolved when we evaluated him at his follow-up appointment 2 weeks later. Notably, daratumumab was not discontinued, as this was a critical medication for our patient’s health. He had also been taking allopurinol and colchicine for nearly a decade for gout, but he had no other chronic medications otherwise.

## Discussion

The pathogenesis of lichenoid drug eruptions remains unclear, but is thought to be T cell-mediated, similar to lichen planus.^7^^,^^8^ A drug-incited event ultimately leads to recruitment of cytotoxic T cells that induce death of keratinocytes. Other chemical mediators like interferon-gamma are known to increase in production in both lichenoid drug eruptions and lichen planus.

Histopathology findings of a lichenoid drug eruption includes a band-like lymphocytic infiltrate obscuring the dermoepidermal junction, with basal keratinocyte vacuolar degeneration, representing an interface/lichenoid dermatitis.^8^ Additional features that are more specific for lichenoid drug eruption include the presence of eosinophils and focal parakeratosis.^7^^,^^8^

The mainstay treatment for lichenoid drug eruption is identification and discontinuation of the causative medication.^7^^,^^8^ After discontinuation, spontaneous resolution typically occurs within weeks to months. Otherwise, there are no well-established treatments, although multiple modalities have been employed for symptomatic management. One review found that the most common treatments included topical steroids (42.1%) and systemic steroids (16.7%).^7^ Other treatments have included antihistamines, topical calcineurin inhibitors, systemic retinoids, and phototherapy. In patients like ours, where causal medication is essential, it may be continued with close monitoring and symptomatic management.

We treated our patient with intramuscular triamcinolone acetonide to gain rapid control of his pruritus. The mechanisms by which triamcinolone acetonide reduces pruritus is not fully understood, though it is generally believed that steroids reduce itch by decreasing inflammation and inhibiting pruritic cytokine cascades (ie IL-4, 13, 31).^9^ When administered intramuscularly, triamcinolone acetonide acts as a depot and provides prolonged systemic anti-inflammatory and anti-pruritic effects.^10^ A 40-80 mg dose can provide effects for more than 12 weeks. This was extremely beneficial for our patient, as one-time treatment alleviated his pruritus within days. This enabled him to continue with daratumumab infusions, and to continue performing his duties as an active-duty soldier without pause, which is paramount when considering the military medical evaluation board process our patients can experience.

In conclusion, we highlight a novel lichenoid drug eruption that responded well to intramuscular triamcinolone acetonide. Our case suggests that immunomodulatory effects of daratumumab over several months may have triggered a T-cell mediated reaction against basal keratinocytes, though more research is needed to elicit precise pathophysiology. We add to the literature a novel case that explores biologics and adverse cutaneous reactions, which further exemplifies how dermatology can contribute to the management of this unique patient population.

### Declaration of generative AI and AI-assisted technologies in the manuscript preparation process

During the preparation of this work the author(s) used OpenEvidence to evaluate and synthesize the most up to date literature regarding lichenoid drug eruptions, daratumumab related cutaneous adverse events, and other relevant sources discussed in the manuscript. After using this tool/service, the author(s) reviewed and edited the content as needed and take(s) full responsibility for the content of the published article.

## Conflicts of interest

None disclosed.

## References

[1] Palumbo A., Chanan-Khan A., Weisel K. (2016). Daratumumab, bortezomib, and dexamethasone for multiple myeloma. N Engl J Med.

[2] Facon T., Kumar S., Plesner T. (2019). Daratumumab plus lenalidomide and dexamethasone for untreated myeloma. N Engl J Med.

[3] Yun X., Zhou Y., Wu D., Liu Y., Wu Q. (2024). A real-world pharmacovigilance study of FDA adverse event reporting system events for daratumumab. Expert Opin Drug Saf.

[4] Wu J., Wu H., Chen L. (2024). Safety of daratumumab in the real-world: a pharmacovigilance study based on FAERS database. Expert Opin Drug Saf.

[5] Kastritis E., Palladini G., Minnema M.C. (2021). Daratumumab-Based treatment for immunoglobulin light-chain amyloidosis. N Engl J Med.

[6] Villarreal-González R., Flores-García N., Cadenas-García D., Gómez-De León A., Piñeiro-Retif R., Vidal-Gutiérrez O. (2025). Daratumumab desensitization: novel approaches in POEMS syndrome experience. J Oncol Pharm Pract.

[7] Maul J.T., Guillet C., Oschmann A. (2023). Cutaneous lichenoid drug eruptions: a narrative review evaluating demographics, clinical features and culprit medications. J Eur Acad Dermatol Venereol.

[8] Cheraghlou S., Levy L.L. (2020). Fixed drug eruptions, bullous drug eruptions, and lichenoid drug eruptions. Clin Dermatol.

[9] Sutaria N., Adawi W., Goldberg R., Roh Y.S., Choi J., Kwatra S.G. (2022). Itch: pathogenesis and treatment. J Am Acad Dermatol.

[10] Shahinfar S., Maibach H. (2023). Enigma of intramuscular triamcinolone acetonide (Kenalog) efficacy. Clin Pharmacokinet.

